# Polo-like kinase 1 suppresses lung adenocarcinoma immunity through necroptosis

**DOI:** 10.32604/or.2023.030933

**Published:** 2023-09-15

**Authors:** PENGCHENG ZHANG, XINGLONG ZHANG, YONGFU ZHU, YIYI CUI, JING XU, WEIPING ZHANG

**Affiliations:** 1 Department of Oncology, The Third Affiliated Hospital of Zhejiang Chinese Medical University, Hangzhou, China; 2 Department of Oncology, Anhui Zhongke Gengjiu Hospital, Hefei, China; 3 Department of Oncology, The First Affiliated Hospital of Anhui University of Chinese Medicine, Hefei, China; 4 Department of Biochemistry and Molecular Biology, School of Integrated Chinese and Western Medicine, Anhui University of Chinese Medicine, Hefei, China

**Keywords:** PLK1, Lung adenocarcinoma, Prognosis biomarker, Immune infiltration, Necroptosis

## Abstract

Polo-like kinase 1 (PLK1) plays a crucial role in cell mitosis and has been associated with necroptosis. However, the role of PLK1 and necroptosis in lung adenocarcinoma (LA) remains unclear. In this study, we analyzed The Cancer Genome Atlas (TCGA) and Genotype-Tissue Expression databases to evaluate the prognostic value and mechanistic role of PLK1 in LA. PLK1 was found to be highly expressed in LA and was positively associated with advanced disease staging and poor survival outcomes. Functional enrichment analysis showed that PLK1 was involved in cell mitosis, neurotransmitter transmission, and drug metabolism. Further analysis using single-sample gene set enrichment analysis and ESTIMATE algorithm revealed a correlation between PLK1 expression and immune infiltration in LA. Silencing of PLK1 using miRNA transfection in LA cells reduced cell proliferation and increased apoptosis, as well as upregulating the expression of necroptosis-related proteins, such as RIPK1, RIPK3, and MLKL. Additionally, nude mouse transplantation tumor experiments demonstrated that silencing PLK1 reduced the growth capacity of LA cells. These findings suggest that PLK1 plays a critical role in LA progression by regulating necroptosis and immune infiltration, and may serve as a potential therapeutic target for immunotherapy. Furthermore, PLK1 expression can be used as a prognostic biomarker for LA patients.

## Introduction

Lung cancer (LC) remains a major global health burden, responsible for a significant proportion of global cancer-associated deaths [1]. LC is mainly classified into small cell lung cancer (SC) and non-small cell lung cancer (NSCLC), which occupying 15% and 85% of the total [2]. Of the NSCLC subtypes, lung adenocarcinoma (LA) is the most frequent, representing approximately 40% of all LC cases [3]. Despite advances in treatment, the 5-year survival rate for advanced LA is still lower than 20% [4,5], mainly due to the low mutation rate at therapeutic loci and the primary resistance of tumors to current therapies, including immunotherapy [6]. So, it is therefore of huge interest to investigate new immunotherapeutic sites and develop new therapeutic strategies for patients with advanced LA. Polo-like kinase 1 (PLK1) is one pivotal regulator of eukaryotic cell division, with a central role in mitosis [7,8].

PLK1 controls various cellular processes during S/G2 and M-phases by phosphorylating specific substrates, including mitosis, centrosome maturation, spindle assembly, sister chromatid cohesion, and cytoplasmic division [9]. Moreover, recent studies have broadened our horizon of the functions of PLK1 beyond cell cycle regulation [10], including autophagy, DNA damage response, apoptosis, as well as cytokine signaling [11,12]. In recent years, PLK1 inhibitors have demonstrated notable tumor reduction in highly proliferative CCND1-driven breast cancer bone metastases [13] and improved oxaliplatin resistance levels in patients with advanced colorectal cancer [14]. However, despite the growing body of evidence highlighting the crucial part of Polo-like kinase 1 (PLK1) in tumorigenesis and tumor progression, studies on the role of PLK1 in lung adenocarcinoma (LA) are limited. Specifically, there is a paucity of studies exploring the association of PLK1 with immune infiltration in LA. Furthermore, although our previous study has verified the involvement of PLK1 in the regulation of necroptosis in LA [15], the exact mechanism by which PLK1 modulates necroptosis in this malignancy remains unknown.

Necroptosis is one form of programmed necrotic cell death [16]. It is mainly under medication of serine/threonine kinase 1 (RIPK1) and RIPK3 and their downstream Mixed lineage kinase domain like (MLKL). RIPK3 phosphorylation is central to the necroptosis pathway, which subsequently gives rise to phosphorylation of MLKL, triggers oligomerization, translocation to the plasma membrane, and leads to membrane permeabilization, ultimately causing cell necrosis. Although the molecular mechanisms of necroptosis have been revealed for many years, its role in tumorigenesis and metastasis has only been reported in recent years. Necroptosis plays different roles in different tumors [17]. In several breast cancer cell strains, knocking down RIPK1, RIPK3 or MLKL genes in cancer cells greatly lowered their tumorigenicity. However, Seifert et al. [18] demonstrated that *in vivo* deletion of RIPK3 or RIPK1 attenuated pancreatic cancer progression as well as immunosuppression in mice. But, the specific mechanism of its role in other tumorigenesis remains to be explored.

**Figure 1:**
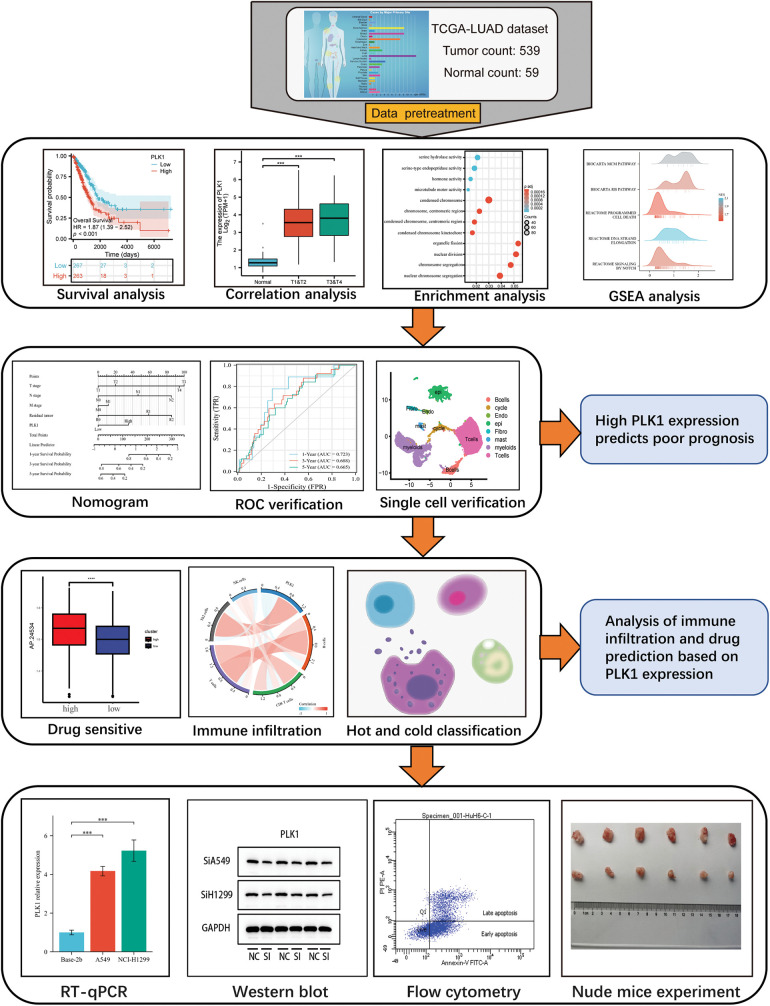
Technology roadmap.

This study assessed the differential expression of PILK1 in LALA and evaluated the prognostic value of PLK1 for LA. Then, the study analyzed the primary pathways of PLK1 differential genes through enrichment analysis and analyzed the association between PLK1 and immune cells to assess its effect on immune infiltration and compare the expression of immune cells in cold and hot tumors. Validated by *in vitro* experiments, we analyzed PLK1 expression in different LA cell lines as well as in normal cells. Subsequently, silencing and overexpression of PLK1 were performed to detect the proliferation level of LA cells, and LA cell apoptosis was determined through flow cytometry, and necroptosis-related pathway protein expression was detected by protein blot analysis. Finally, the impact of silencing PLK1 on the growth of transplanted tumors was explored via transplantation tumor assay in nude mice. The final results indicated that PLK1 is a potent prognostic biomarker for LA and that PLK1 probably inhibits tumor immune infiltration and thus promote proliferation and metastasis of LA cells through inhibiting necroptosis (Fig. 1).

## Materials and Methods

### Data collection

Pan-cancer data were acquired from UCSC XENA (https://xenabrowser.net/datapages/) and LA data were downloaded from The Cancer Genome Atlas (TCGA) and screened for 535 samples totally with clinical information. GSE123902, GSE31210 were acquired from the NCBI database (http://www.ncbi.nlm.nih.gov/). GSE31210 is a transcriptomic dataset consisting of 226 samples of LA. On the other hand, GSE123902 comprises a single-cell dataset containing 13 samples of LA *in situ* and metastatic samples, along with 4 non-tumor samples.

### Expression of PLK1 in lung adenocarcinom patients

The 535 LA samples were assigned to high PLK1 expression/low expression group in the light of the median PLK1 expression value. Due to the data type being counts data, we chose to perform the analysis using Deseq2 [19] R package for analyzing the inter-group differences, with log fold change absolute values >1.5 and *p*-value < 0.05 as threshold parameters. Through the ggplot2 (v3.3.3) R package, volcano and heat maps of the differential genes were visualized Human Protein Atlas (HPA) (http://www.proteinatlas.org), HPA051638 (Sigma-Aldrich), HPA053229 (Sigma-Aldrich) antibodies were used for verification about the expression of PLK1 protein levels in normal tissues *vs*. LA tissues.

### Survival analysis and construction of nomogram

Survival analysis was conducted in this study (the cut-off value: median PLK1 expression), and the risk factors were visualized with the pheatmap R package. The survival package (v3.2-10) was adopted for the statistical analysis of survival data [20], and the survival curves of the high and low-risk groups were plotted using the survminer R (v0.4.9) package. With the Kaplan–Meier method, the survival curves were drawn, and the statistical significance was calculated via the log-rank test. The accuracy of the time-dependent receiver-operating characteristic (ROC) curve was evaluated via the timeROC [21] package (v1.17.0.1), and the AUC area was calculated for 1, 3, and 5 years. For validating the predictive ability of the model, the GSE31210 dataset was used, and survival curves for high and low-risk groups, ROC curves for 1, 3, and 5 years, ROC curves based on clinical traits, and correlations of clinical traits in high and low-risk groups were also drawn based on the validation set. With multifactorial cox analysis, independent prognostic factors were identified, and one nomogram was created using RMS (v6.2-0) for prediction of the overall survival (OS) probability. The performance of the nomogram was evaluated using calibration plots.

### Functional enrichment analysis of high and low risk groups

Through the clusterProfiler [22] R package (v3.14.3) to carry out functional enrichment of DEGs, including Gene Ontology (GO) and Kyoto Encyclopedia of Genes and Genomes [23] (Kyoto Encyclopedia of Genes and Genomes, KEGG), and *p* < 0.05 for the False discovery rate (FDR) was considered greatly significant. And the gene set “c2.cp.v7.2.symbols.gmt” was downloaded from MSigDB database and Gene Set Enrichment Analysis (Gene Set Enrichment Analysis) was carried out, and *p*.adjust < 0.05 indicated significant enrichment.

### Protein-protein interaction (PPI) network analysis

The protein-protein interaction (PPI) network of differentially expressed genes in both high and low-risk groups was constructed using the STRING database (http://string-db.org). The following parameters were set: correlation coefficient of 0.4, abs(Log2 FC) > 1, k-means clustering with a quantity of 4, and disconnected nodes were hidden in the network. The PPI results were exported from the STRING database and visualized by Cytoscape. In addition, we analyzed the Hub genes in the PPI network using the MOCODE plugin.

### Gene-related network and drug prediction

A comprehensive analysis of gene regulation and drug prediction was conducted using state-of-the-art computational methods. Firstly, miRNet [24] database was utilized to construct a hub gene-miRNA regulatory network. Next, the NetworkAnalyst25 database was employed to match the hub genes with their corresponding transcription factors (TFs) to construct a hub gene-TF regulatory network. The resulting networks were visualized using the Cytoscape software.

To further investigate the differential drug response between high and low groups, drug predictions were performed using the pRRophetic package [25], followed by visualization using the ggplot2 package.

### Single cell sequencing analysis

Download LA single cell dataset GSE123902 from GEO database [26]. We applied the “PercentageFeatureSet” to filter cells based on specific criteria, including gene expression exceeding 300, mitochondrial gene expression below 15%. Subsequently, the merged ScRNA-seq data were normalized. The top 2000 highly variable genes were identified using the “FindVariableFeature”. We then scaled all genes using the “ScaleData” and performed dimensionality reduction on the selected 2000 highly variable genes using the “RunPCA” for principal component analysis. Batch correction was performed using the Harmony algorithm. Cell clustering was conducted using the “FindNeighbors” and “FindCluster” with a resolution of 0.8 to identify distinct cell clusters. Furthermore, the UMAP method was employed for additional dimensionality reduction. Finally, the “FindAllMarkers” was utilized to identify 19 clusters based on marker genes. PLK1 expression was visualized using the “Featureplot”, and “Pseudotime” analysis was conducted using the Monocle [27] package.

### Immuno-infiltration analysis

The level of immune infiltration of 24 immune cells was evaluated using the ssGSEA algorithm and the relative enrichment score of these immune cells in LA was evaluated through single sample GSEA. And using the ESTIMATE24 R package to calculate Stromal score, Immune score, and Estimate score to assess PLK1 and immune correlation. And based on the median Immune score to classify LA into cold and hot tumors, the differential expression of PLK1 in cold and hot tumors was plotted using the ggpubrR package, and based on GSVA [28] (v1.34.0) R package was analyzed to obtain a heat map of differential expression between cold and hot tumors.

### Cell culture and transfection

Bease-2b, A549, and NCI-H1299 cells (ATCC, Virginia, USA) were obtained from the Laboratory of Molecular Biology of Tumors, Southeast University. All cell lines were authenticated in the laboratory and log-phase cells from the 4th to 5th passages were used for subsequent experiments. The cells were maintained in RPMI-1640 (HyClone, Guangzhou, China) medium having 100 μM streptomycin, 100 U/mL penicillin as well as 10% Fetal bovine serum (FBS, HyClone®, Melbourne, Australia). Cells were subjected to incubation (5% CO_2_, 37°C) in a humidified atmosphere.

Bease-2b, A549 as well as NCI-H1299 cells were inoculated in 6-well plates, followed by transfection with small interfering RNA (miRNA) (Beyotime Shanghai, China) targeting PLK1 (Table 1). For negative control (Si-NC) plasmids, knockdown plasmids, overexpression plasmids, and empty controls, cells were transfected with Lipofectamine® 2000 (Invitrogen, San Diego, USA) under the manufacturer’s instructions. Cells were treated by 6-h incubation with serum-free medium containing 10% FBS after transfection and then given 24–72 H incubation to select stably transfected cells for subsequent experiments. In the experimental design for the A549 and H1299 cell lines, both experimental groups were treated with SiRNA-1 and SiRNA-2, respectively, while the control group was treated with Si-RNANC. Each group was replicated three times to ensure the reliability of the results.

**Table 1:** The primer sequences employed in RT-qPCR

Primer name	Primer sequence (5′–3′)
PLK1 forward	AAAGAGTCCCGGAGGTCCTA
PLK1 reverse	GGCTGCGGTGAATGGATATTTC
Si PLK1 forward	TATACGATACAAGGCTGTTAGAGAG3
Si PLK1 reverse	ACTGTGGGCGATGTGCGCTCTG
GAPHD forward	CGAGCCACATCGCTCAGACA
GAPHD reverse	GTGGTGAAGACGCCAGTGGA

### Real-time quantitative PCR

Total RNA was isolated from tissues and cells through TRIzol reagent. cDNA was acquired through reverse transcription with TaKaRa Ex Taq® (Takara, Beijing, China) and gene expression was identified using PrimeScript® RT-PCR Kit II (Takara, Beijing, China). The data were calculated via the 2^ΔΔCq^ method with GAPDH as control gene for normalization, and each group of experiments was conducted repeatedly three times. The primer sequences designed are listed in Table 1.

### CCK-8 detection

After the cells had reached 90% confluence in culture, the Bease-2b, A549 and NCI-H1299 cells transfected with Si-PLK1 were inoculated into 96-well culture plates at 4000 cells per well, with four replicate wells for each group. They were then subjected to incubation (37°C) in a 5% CO_2_ incubator and tested for 450 nm absorbance using 10 μL CCK-8 (Bimake, Texas, USA) at 24, 48, and 72 h, and each group of experiments was conducted repeatedly three times.

### Apoptosis detection by flow cytometry

Cells were seeded at a density of 100,000 cells/well in a 6-well plate. After 72 H of incubation, the corresponding groups were retrieved from the incubator. The cells were then collected and washed twice with pre-chilled PBS. Subsequently, each group was treated separately with 5 μL of Annexin V/FITC (Acmec, Shanghai, China) for 5 min. Following the treatment, the samples were analyzed using flow cytometry. For each group, the experiment was repeated three times to ensure statistical reliability. The collected data were subjected to statistical analysis to obtain meaningful results. Throughout the entire procedure, the cells were handled on ice to maintain their viability. To prepare the binding solution, the buffer was diluted with water at a ratio of 1:9, while for the experimental group, PE dye and 7-AAD dye were added along with the binding solution. After a 15-min incubation, the cells were gently mixed, filtered, and transferred to new centrifuge tubes before flow cytometry analysis. The described protocol was designed to accurately assess and analyze the cells’ characteristics.

### Western blot

For verifying the role of PLK1 in necroptosis, the expression levels were analyzed through Western blot. LA cells were lysed by RIPA-containing protease inhibitors (Thermo Scientific, Waltham, USA), and total proteins were obtained through separating the lysates by sodium dodecyl sulfate-polyacrylamide gel electrophoresis (SDS-PAGE, Thermo Scientific, Waltham, USA) and blotted on polyvinylidene difluoride (PVDF) membranes for analysis. Antibodies to RIPK1, RIPK3, and MLKL proteins were added for final protein quantification. Protein blotting analysis was performed using ImageJ software (version 1.8.0, Bethesda, MD, USA).

### Nude rat experiments

For the *in vivo in situ* xenograft implantation assay, female LA nude mice (6 weeks old, weighing 16–20 g) were assigned to 2 groups (n = 6 per group) in a random way; PLK1 knockdown and control groups. Stably transfected LA cells were suspended in 100 μL PBS + 100 μL Matrigel substrate, followed by injection into the axilla of nude mice at 5 × 105 cells. Tumor volumes were measured every 3 days using the formula: length × width × 0.5 (cm^3^). 24 days after injection, the mice were sacrificed, and the tumors were removed and weighed. The tumor volume was calculated, and the tumor growth curve was plotted. The ethical review board (IRB) authorized animal experimentation (AHUCM mouse-2022059).

### Statistical analyses

This study conducted data analyses through R software (4.1.3), with results expressed as the mean ± standard deviation, measures are shown as percentages. Chi-Square test Student’s *t*-test was adopted for comparing the inter-group difference, while one way analysis of variance (ANOVA) was adopted. The Wilcoxon rank-sum test and logistic regression were used for assessing the correlations between clinical features and PLK1 Spearman was used for correlations and all *p*-values were bilateral. *p* < 0.05 implies a notable difference.

## Result

### PLK1 is highly expressed in lung adenocarcinoma species

**Figure 2:**
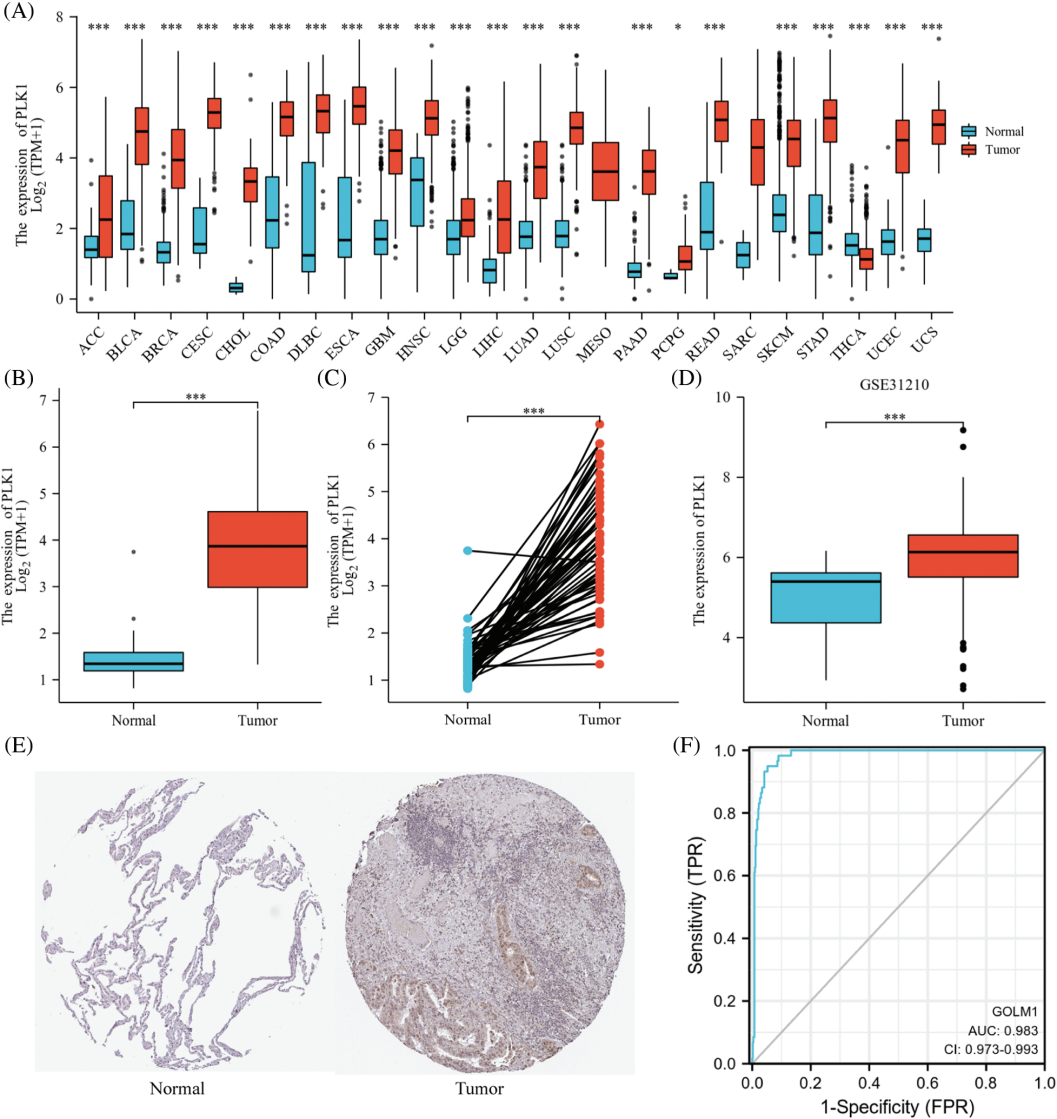
PLK1 expression differences. (A) PLK1 expression differences in different tumors; (B) PLK1 expression differences between tumor tissues and normal tissues in TCGA and GTEx databases; (C) PLK1 expression differences between cancer and paraneoplastic tissues in paired samples; (D) PLK1 expression differences between LA and normal tissues in GSE31210; (E) PLK1 protein expression differences between LA and normal tissues in HPA database; (F) ROC curves of LA tissues and normal lung tissues in TCGA database. Expression differences; (F) ROC curves of LA tissues from TCGA database with normal lung tissues for classification. *p < 0.05, ***p < 0.001.

PLK1 gene was highly expressed in various cancers, including bladder uroepithelial carcinoma, hepatocellular carcinoma, LA as well as gastric adenocarcinoma (Fig. 2A). Meanwhile, according to the ROC curve, PLK1 was a favorable predictor of LA compared to other tumors (AUC = 0.983, 95% CI = 0.973–0.993) (Fig. 2F). In unpaired samples, PLK1 expression was significantly higher in LA cells than in normal lung tissue (*p* < 0.001) (Fig. 2B). In LA cancer and paracancer paired samples, LA expression was similarly higher than in paracancer tissue (*p* < 0.001) (Fig. 2C) (See Suppl. Table 1 for a table of patient baseline information). Furthermore, we validated PLK1 expression using the validation set GSE31210 and uncovered higher PLK1 expression in tumor tissues than that in normal ones (Fig. 2D). PLK1 protein expression in different lung tissues via the HPA database revealed greatly higher levels of LA protein expression (Fig. 2E).

### PLK1 correlation with clinical characteristics and survival analysis

**Figure 3:**
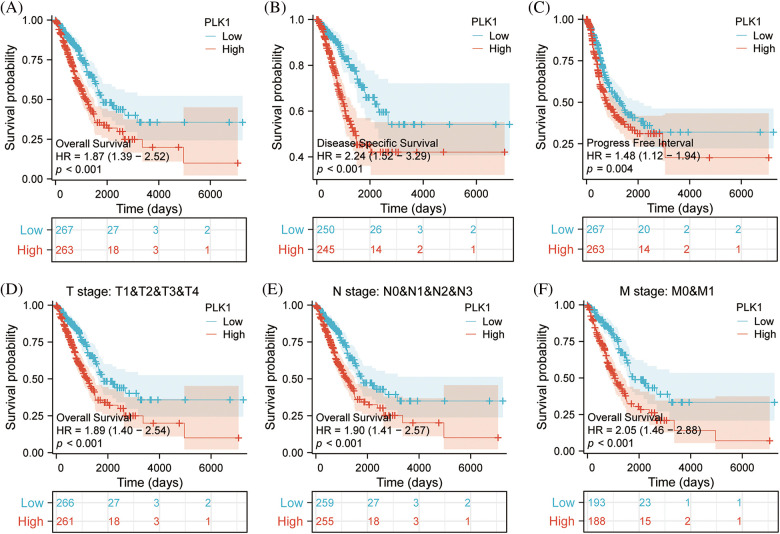
Effect of PLK1 expression differences on survival prognosis. (A) Patients with high expression of PLK1 have significantly poorer overall survival (OS) compared to those with low expression in LA (*p* < 0.001); (B) patients with high expression of PLK1 have significantly poorer disease specifitic survival (DSS) compared to those with low expression in LA (*p* < 0.001); (C) patients with high expression of PLK1 have significantly poorer disease free intervals (DFI) compared to those with low expression in LA (*p* < 0.001); (D–F) patients with high PLK1 expression have worse outcomes across all stages of TNM classification compared to those with low expression in lung adenocarcinoma (*p* < 0.001). ***p < 0.001.

According to survival analysis, patients in the PLK1 high expression group had worse prognosis for OS (HR = 1.87, 95% CI = 1.39–2.52, *p* < 0.001), DFS (HR = 2.24, 95% CI = 1.52–3.29, *p* < 0.001), PFI (HR = 1.48, 95% CI = 1.21–1.94, *p* = 0.005) had a unfavorable prognosis (Figs. 3A–3C). Meanwhile, the T-stage (HR = 1.89, 95% CI = 1.40–2.54, *p* < 0.001), N-stage (HR = 1.90, 95% CI = 1.41–2.57, *p* < 0.001) M-stage (HR = 2.05, 95% CI = 1.46–2.88, *p* < 0.001) of PLK1 high expression group patients’ prognosis poorer (Figs. 3D–3F), also indicating the detrimental effect of high PLK1 expression on patient prognosis. Meanwhile, according to clinical correlation analysis, PLK1 gene was significantly expressed in LA tissues compared with normal tissues in all TNM stages (*p* < 0.001) (Suppl. Figs. 1A–1C). The pathological stages of patients showed that PLK1 expression was higher in LA tissues than in normal tissues (*p* < 0.001). Furthermore, stages III–IV patients had greatly higher PLK1 expression than patients in other stages (*p* < 0.01) (Suppl. Fig. 1D). Analysis of patients’ residual tumor showed that PLK1 expression was greatly higher in tissues with residual tumor (*p* < 0.001) (Suppl. Fig. 1E). And PLK1 expression was higher in patients who smoked than in non-smokers (*p* < 0.01) (Suppl. Fig. 1F).

### Identification and functional enrichment of DEGs for LA

**Figure 4:**
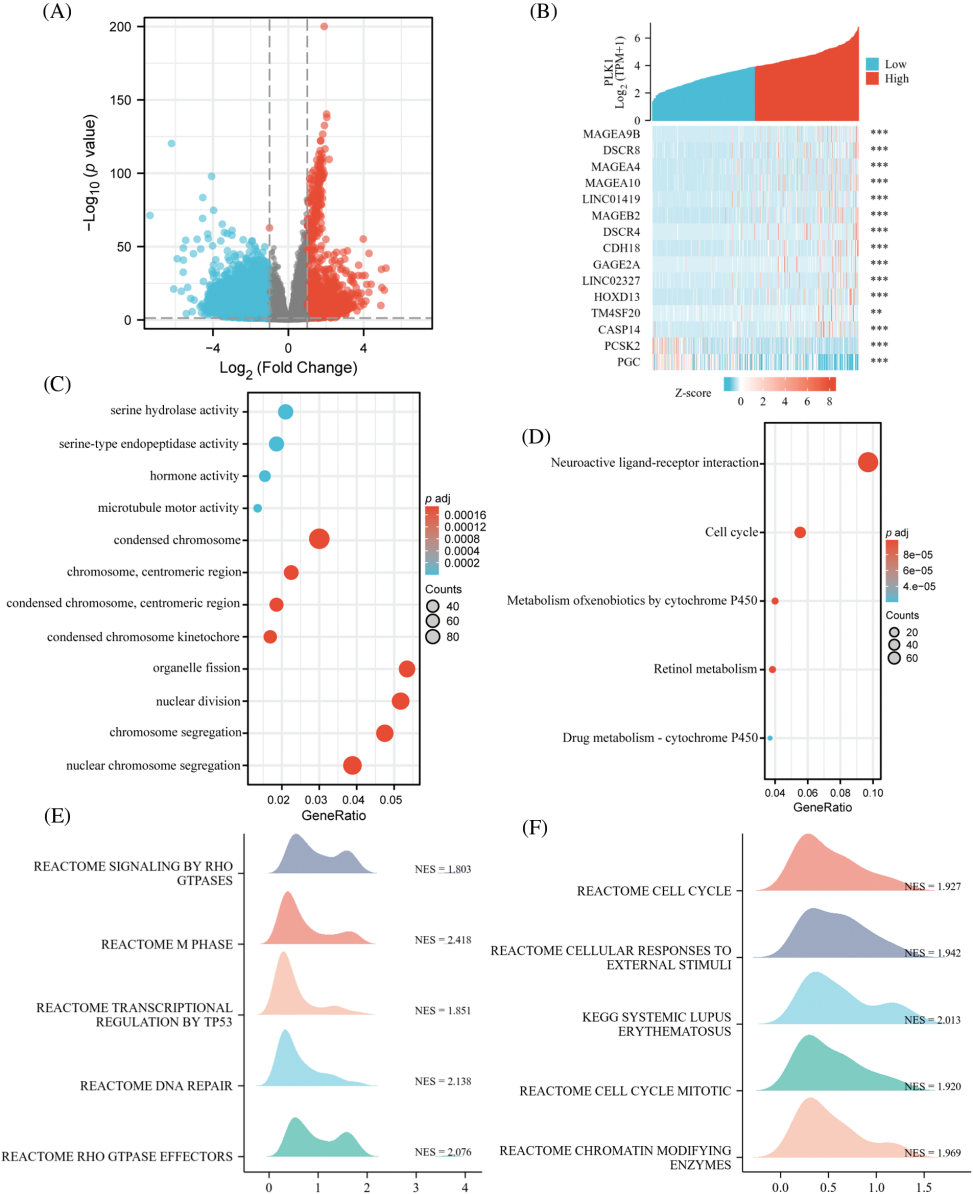
Identification and functional enrichment of DEGs. (A) Volcano map of DGEs; (B) co-expression heat map of top 15 genes; (C) GO pathway enrichment. The representative bar chart showing the items enriched in Biological Process (BP), Cell Component (CC), and Molecular Function (MF); (D) KEGG pathway enrichment. (E, F) GSEA enrichment. **p < 0.01, ***p < 0.001.

In TCGA, total of 3247 each differential genes were detected, including 2085 up-regulated genes, and 1162 down-regulated ones (Fig. 4A) (abs (log2FoldChange) top 500 differential genes in Suppl. Table 2). And 13 up-regulated genes and 2 down-regulated ones were found in the top of 15 genes (Fig. 4B). Enrichment analysis was conducted for gaining insight into the biological function of these genes. We found that the differential genes were enriched in pathways involved in necroptosis, like Serine hydrolase activity, in addition to mitosis-related functions, indicating that PLK1 may play a role in regulating the biological process of LA necroptosis. Moreover, subsequent KEGG analysis (Fig. 4D) (Suppl. Table 3) revealed enrichment for pathways such as Metabolism of xenobiotics by cytochrome P450, implying the possible implication of PLK1 in the regulation of drug resistance in tumor cells, and Neuroactive ligand-receptor interaction, indicating the implication of PLK1 in tumor neurotransmission and the biology of messaging (Suppl. Table 4). GSEA analysis further corroborated the GO/KEGG enrichment results, while enrichment in RHO gtpases also indicated the involvement of PLK1 in multiple processes in tumour growth, including mitosis, cell death, immunosuppression, and drug resistance (Figs. 4E and 4F).

### PPI network analysis and screening of hub genes

**Figure 5:**
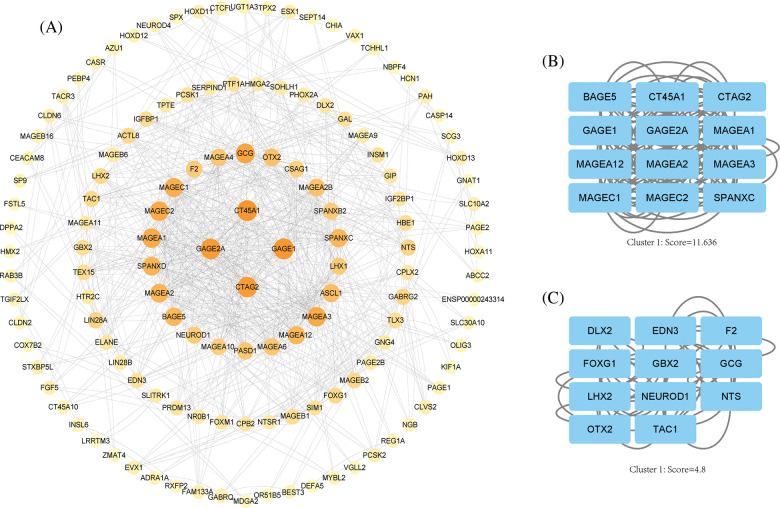
PPI network analysis. (A) Cytoscape software shows the PPI network, the darker the color, the higher the correlation. (B) Hub gene module 1, score value = 11.636. (C) Hub gene module 2, score value = 4.8.

We imported differential genes into STRING database to obtain the PPI network of differential genes (Fig. 5A) and acquired the central genes as CT45A1, GAGE1, GAGE2A, CTAG2, MAGC1, MAGC2, MAGA1. These genes are mainly highly expressed in tumors and involved in several processes such as tumor proliferation. Finally, we obtained Hub gene modules by cytoscape plugin MOCODE and got 5 modules, the 2 largest modules with score values of 11.636 and 4.8, respectively (Figs. 5B and 5C).

### Prognostic value of PLK1 in LA

**Figure 6:**
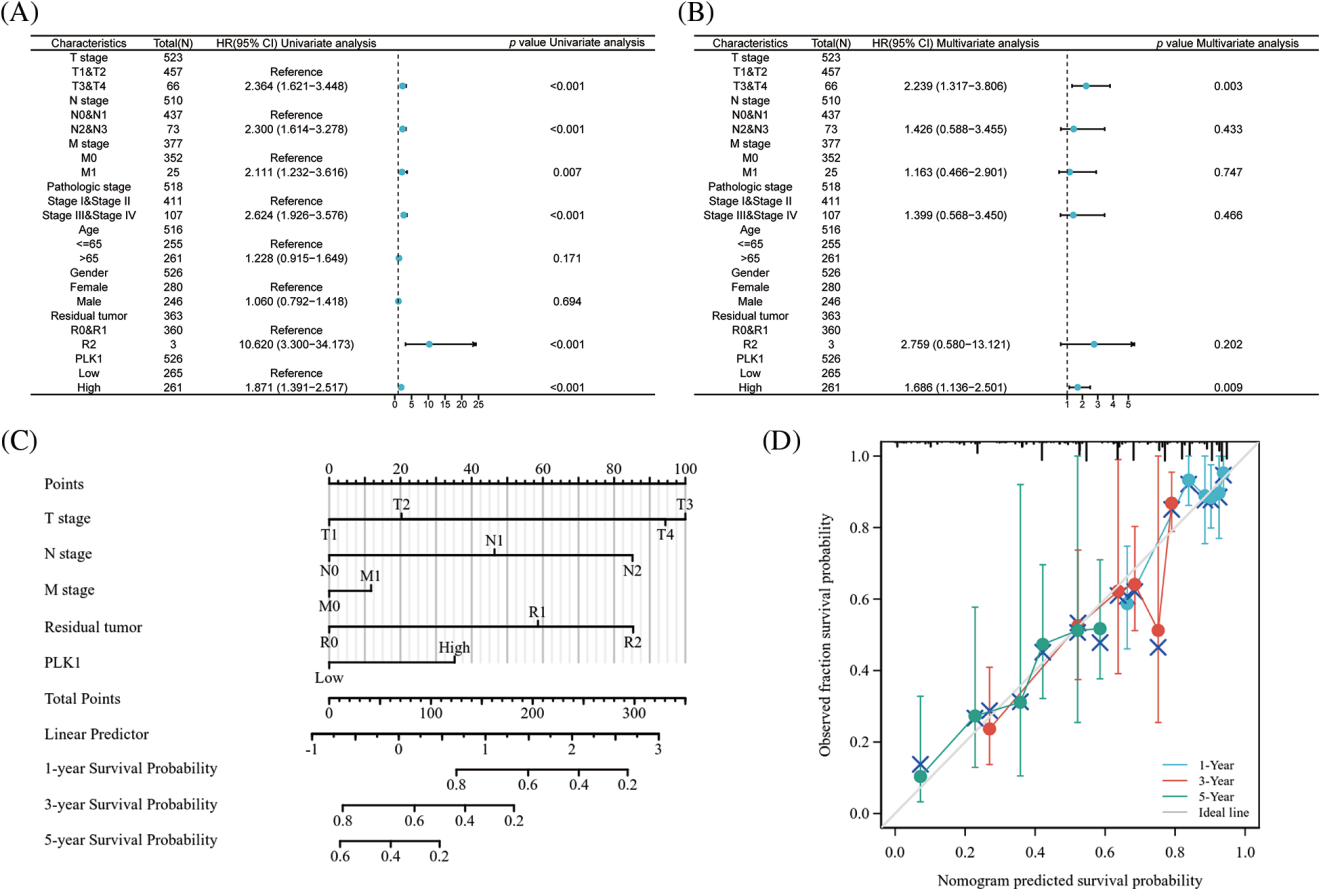
Prognostic value of PLK1 in LA. (A) Forest plot of single-factor cox regression analysis; (B) forest plot of multi-factor cox regression analysis; (C) nomogram for predicting 1, 3, and 5 overall survival of LA patients; (D) calibration curve of columnar line plot for predicting 1, 3, and 5 overall survival of LA patients.

To verify whether PLK1 could be an independent prognostic factor for LA, we first performed univariate cox regression analysis of high and low PLK1 expression with clinical traits according to median values, and forest plots showed that PLK1 differential expression (*p* < 0.001), TNM stage (*p* < 0.001), Pathologic stage (*p* < 0.001), and Residual tumor (*p* < 0.001) were associated with OS (Fig. 6A), and we then continued these four variables in a multifactorial cox regression analysis, which showed that PLK1 (*p* = 0.009) and tumor TNM stage (*p* = 0.003) independently of other clinical traits as independent prognostic molecules could forecast the patients’ OS (Fig. 6B) (Suppl. Tables 5 and 6). Lastly, we constructed a line graph based on the independent factors of OS. On the Nomogram, the higher the score, the worse the prognosis. The results indicated that PLK1 expression level could be used as a LA-independent predictor (Fig. 6C). Correction curves were used to assess the predictive effect of the column line graph, and the results showed that the column line graph was applicable (Fig. 6D).

### Data set validation

**Figure 7:**
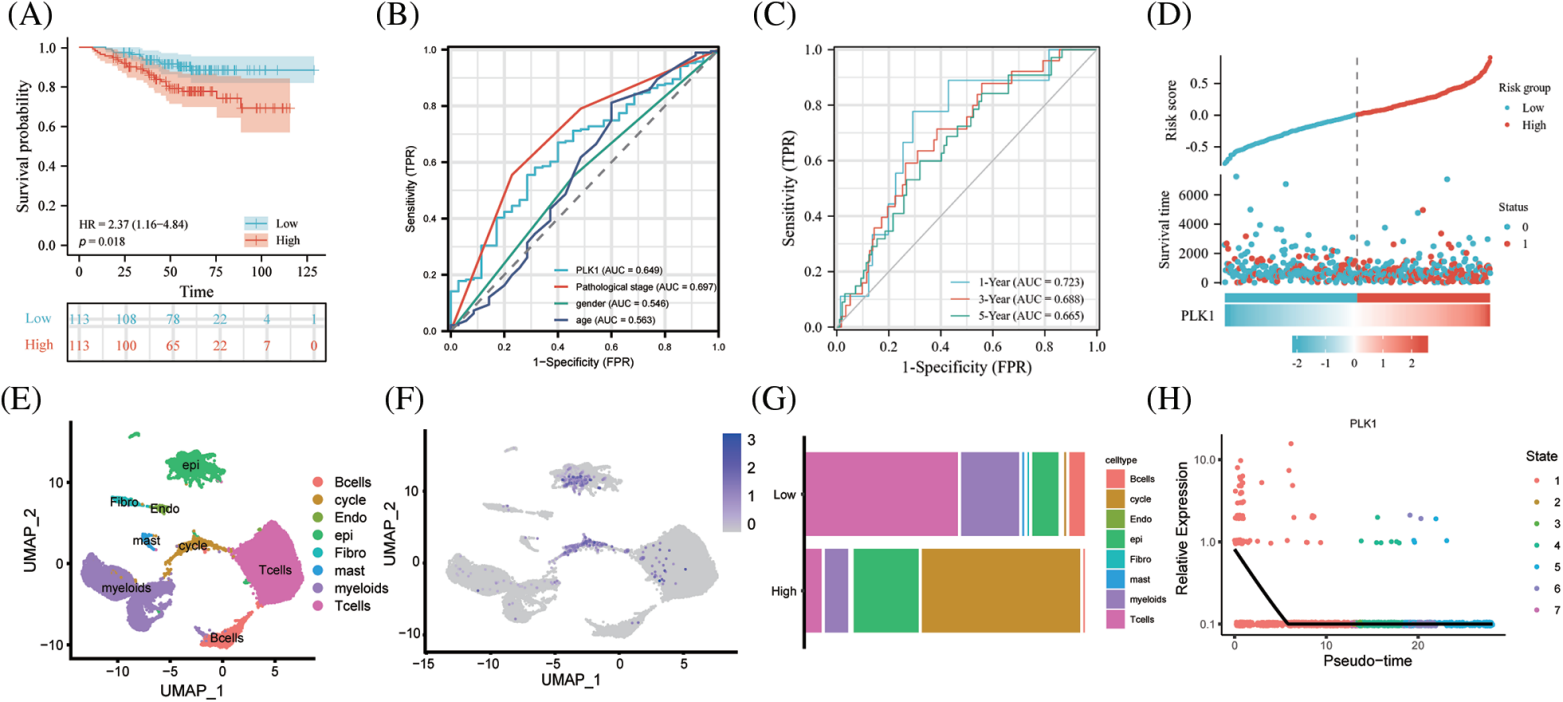
Validation of the GEO dataset. (A) Survival analysis of high- and low-risk groups in the GES31210 validation dataset. Red indicates high risk group, green indicates low risk group. (B) AUC area of the validation set for PLK1 high and low expression combined clinical traits. (C) AUC area of the validation set at 1, 3, and 5 years. (D) PLK1 high and low expression risk factor scores; (E) Umap of GSE123902; (F) Umap of PLK1 expression abundance in different celltype; (G) cellular differences between high and low PLK1 expression groups. (H) Monocle analysis of PLK1 expression.

Based on the prediction of TCGA data, we calculated K-M curves in GSE31210 dataset and found greatly worse prognosis of the high PLK1 expression group than that of the low expression group (HR = 2.37, 95% CI = 1.16–4.84, *p* = 0.018) (Fig. 7A). According to time-dependent ROC analysis, the AUC values at 1, 3, and 5 years were 0.723, 0.668, and 0.665, respectively (Fig. 7B), indicating the applicability of the accuracy of our constructed model. The ROC analysis of clinical traits age, gender, and tumor stage in the combined GSE31210 dataset showed that the AUC of risk score was 0.649, which also demonstrated the guiding effect of PLK1 on the prediction of patient prognosis (Figs. 7C and 7D). We then calculated the risk factor map in LA based on high and low PLK1 expression, and the results also corroborated with the previous.

Subsequent investigations utilizing the scRNA-seq dataset GSE123902 allowed for a more comprehensive examination of PLK1 expression at the single-cell level. Intriguingly, our results unveiled a distinct enrichment of PLK1 within both epithelial cells and cycling cells (Figs. 7E and 7F). Subsequently, based on the median PLK1 expression, cells were classified into high and low expression groups. Strikingly, a comparative analysis of these groups’ cellular compositions revealed a noteworthy reduction in the proportion of immune cells within the high expression group when contrasted with the low expression group. Simultaneously, a substantial elevation in the abundance of epithelial cells and cycling cells was observed within the high expression group. These findings not only hint at a putative role of PLK1 in orchestrating the tumor immune microenvironment but also establish its pronounced enrichment within the tumor milieu (Fig. 7G). Finally, by embarking on a temporal analysis, we gained valuable insights into the temporal dynamics of PLK1 expression, which primarily manifested during the early stages of cellular development. Such observations shed light on the underlying biological mechanisms through which PLK1 governs cellular proliferation (Fig. 7H).

### PLK1 correlates with immune infiltration and immune differences in hot and cold tumors

**Figure 8:**
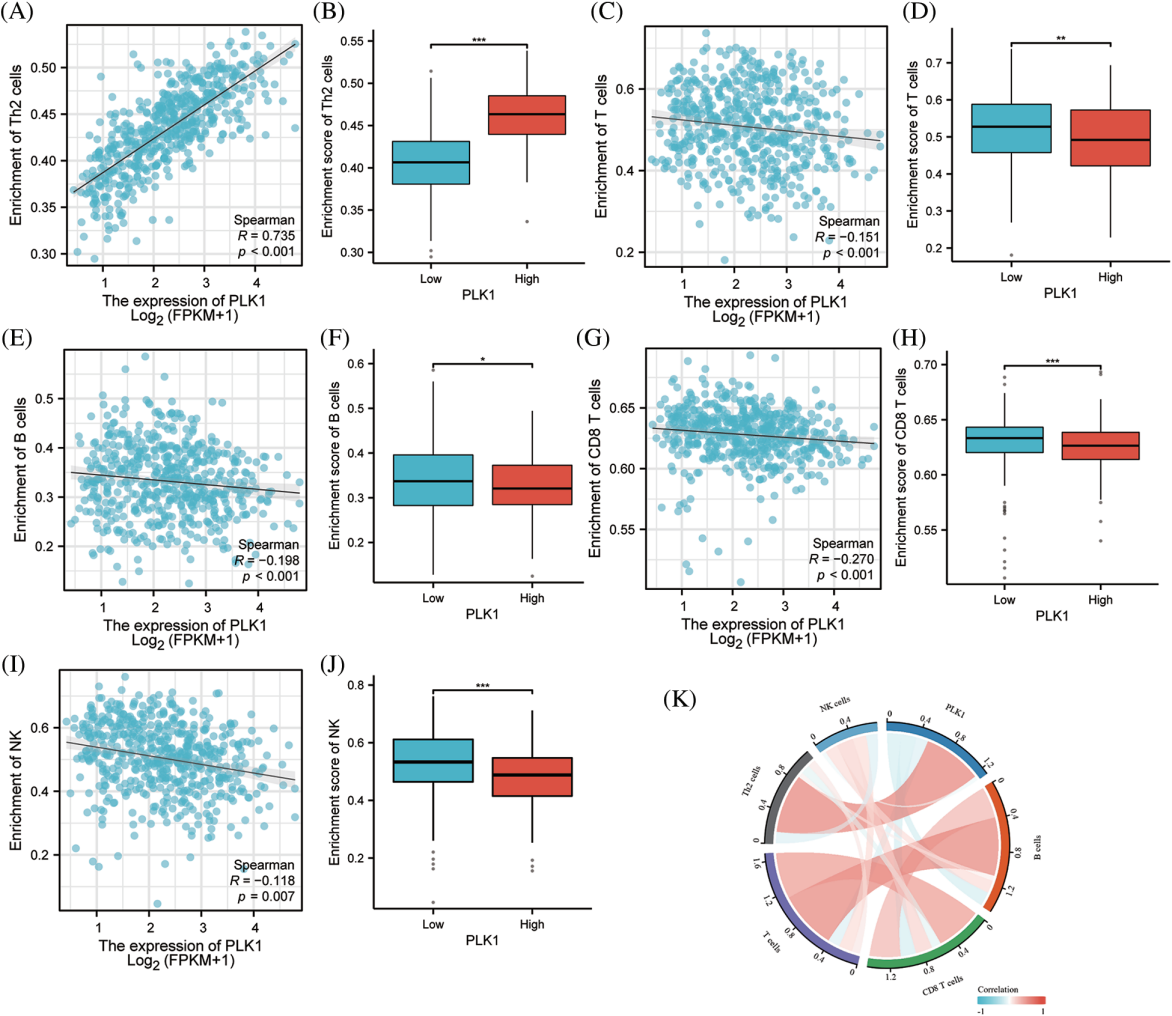
PLK1 on immune infiltration subgroup analysis. (A) PLK1 expression was positively correlated with Th2; (B) PLK1 high expression group was higher than low expression group Th2 cells; (C) PLK1 expression was negatively correlated with T cells; (D) PLK1 high expression group was lower than low expression group T cells; (E) expression group lower expression of B cells than low expression group; (F) PLK1 high expression group was higher than low expression group B cells; (G) PLK1 expression negatively correlated with CD8+ T cells; (H) PLK1 high expression group lower expression of CD8+ T cells than low expression group; (I) PLK1 expression negatively correlated with NK cells; (J) PLK1 high expression group lower expression of NK cells than low expression group; (K) PLK1 correlation with immune cells and chord diagram. *p < 0.05, **p < 0.01, ***p < 0.001.

PLK1 had notable positive correlation with Th2 cells (r = 0.735, *p* < 0.001) by ssGSEA analysis, while it had negative correlation with T cells (r = −0.151, *p* < 0.001), B cells (r = −0.198, *p* < 0.001), CD8+ T cells (r = −0.270, *p* < 0.001), NK cells (r = −0.118, *p* = 0.007) had negative correlated with the major immune cells (Suppl. Table 7). Meanwhile, we performed an immune infiltration subgroup analysis based on the high and low PLK1 expression groups and demonstrated that the immune infiltration scores were higher in the low PLK1 expression group than in the high PLK1 expression group (*p* < 0.05), except for Th2 cells, indicating the suppressive effect of PLK1 on immunity (Figs. 8A–8J). It was also demonstrated by chordal plots that PLK1 was negatively correlated with all immune cells other than Th2 cells (Fig. 8K). This suggests that PLK1 significantly suppresses the immune function of LA patients, which also sets the stage for tumour growth and metastasis. Then, We explored the correlation between PLK1 and immune checkpoints. PLK1 was significantly correlated with most immune checkpoints, with the most significant correlations with CD274, LAG3, CD724, PVRL2, LGALS9, KDR (Suppl. Fig. 2A), suggesting that PLK1 is closely related to LA tumor immunity and has potential as a therapeutic target for LA. At the same time, we found that PLK1 expression was significantly negatively correlated with RIPK3 (r = −0.169, *p* < 0.001) (Suppl. Fig. 2B), which also verified that PLK1 may inhibit necroptosis.

**Figure 9:**
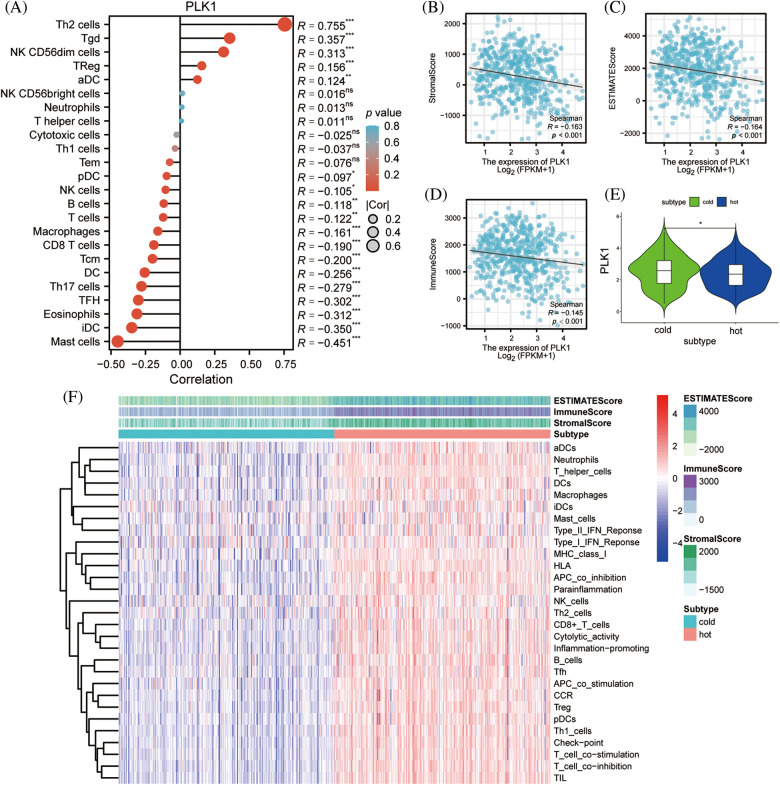
Effect of PLK1 on immune infiltration. (A) Correlation of PLK1 expression with the relative abundance of 24 immune cells. Point sizes correspond to the absolute values of Spearman correlation coefficients. (B) Stromal score; (C) estimate score; (D) immunes core; (E) differences in PLK1 expression in hot and cold tumors; (F) heat map of immune infiltration in hot and cold tumors. *p < 0.05, **p < 0.01, ***p < 0.001.

To clarify the association of PLK1 with immune infiltration, PLK1 expression was notably negatively associated with Stromal score, Immunes score, as well as Estimate score based on the ESTIMATE algorithm (Figs. 9B–9D), demonstrating that PLK1 promotes tumor progression mechanism may be strongly bound up with the inhibition of immune infiltration (Fig. 9A). According to the above scores, we classified them into hot and cold tumors. By difference analysis, we found that PLK1 expression was higher in cold tumors compared with hot tumors (*p* < 0.05) (Fig. 9E), and found that cold tumors were generally low in immune cells expression compared with hot tumors (Fig. 9F). This also demonstrates that PLK1 affects the tumor immune microenvironment by suppressing immune infiltration.

### Prediction of drug sensitivity

**Figure 10:**
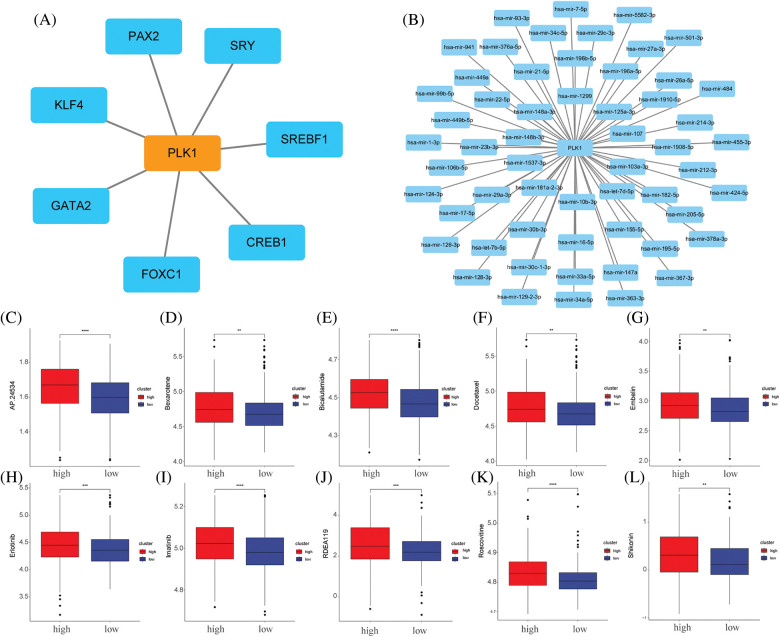
Network construction and drug sensitivity prediction. (A) TF network construction; (B) miRNA network construction; (C) AP.24534; (D) bexarotene; (E) bicalutamide; (F) docetaxel; (G) embelin; (H) erlotinib; (I) imatinib; (J) RDEA119; (K) Roscovitine; (L) Shikonin. **p < 0.01, ***p < 0.001, ****p < 0.0001.

We analysed the transcription factor regulatory network of the PLK1 gene (Fig. 10A), and subsequently we constructed a PLK1-based miRNA regulatory network (Fig. 10B). We then gave high and low expression groups for drug prediction, and the results showed that the PLK1 high expression group was less sensitive to the effects of various therapeutic drugs, suggesting that PLK1 may be the main cause of LA drug resistance (Figs. 10C–10L).

### Effect of PLK1 on proliferation, apoptosis and necrotic apoptosis of LA cells

**Figure 11:**
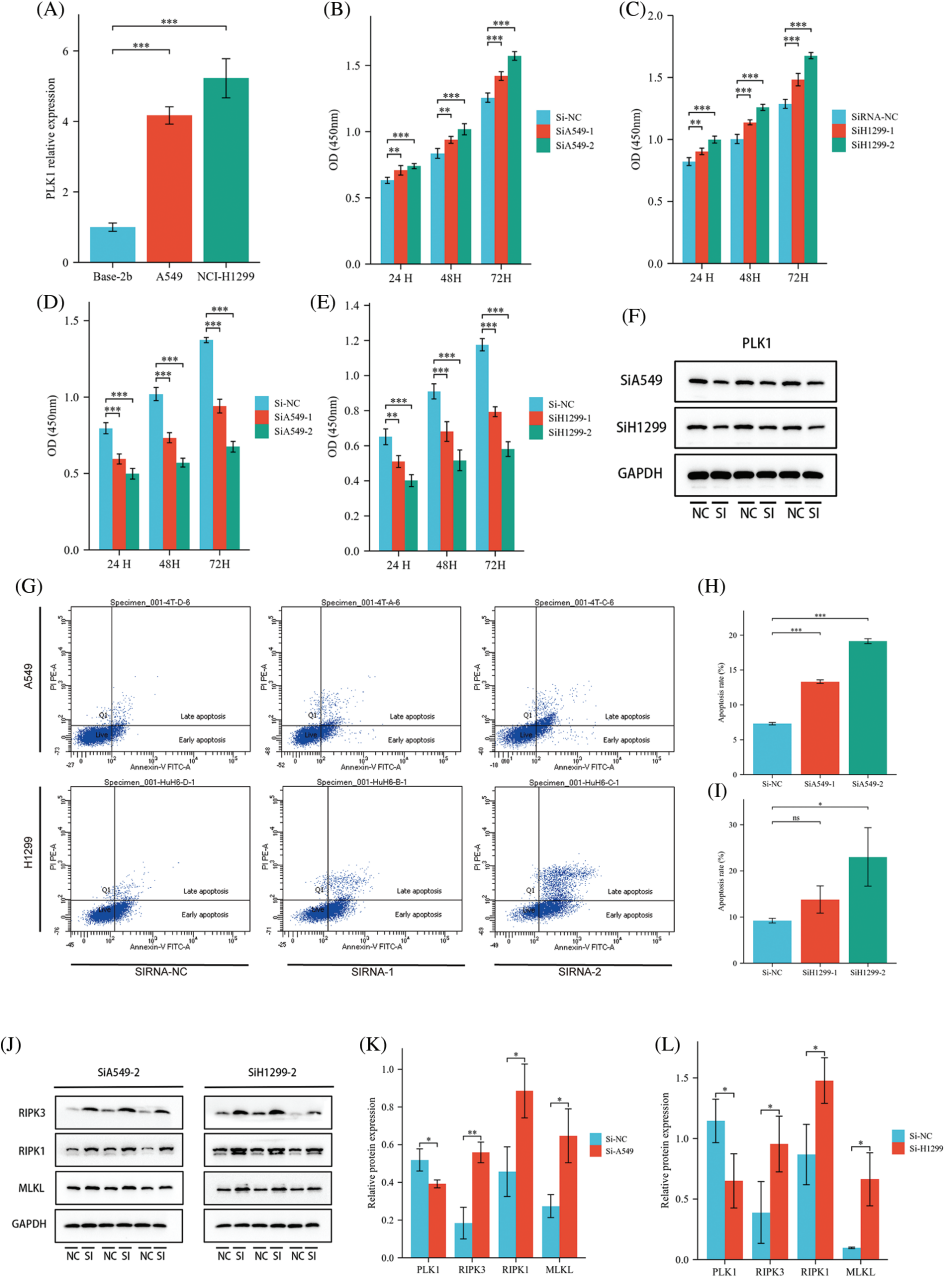
Effect of PLK1 on LA cells. (A) RT-qPCR to verify the expression status of PLK1 in LA cells; (B) effect of overexpression of PLK1 on the absorbance of A549 cell line; (C) effect of overexpression of H1299 on the absorbance of A549 cell line; (D) effect of silencing PLK1 on the absorbance of A549 cell line; (E) effect of silencing PLK1 on the absorbance of H1299 cell line; (F) protein blot to verify PLK1 silencing efficiency; (G) flow cytometry detection of apoptosis level in LA cell line after PLK1 silencing; (H) A549 apoptosis rate; (I) H1299 apoptosis rate; (J–L) protein blotting detection of heavy RIPK3, RIPK1, MLKL expression levels in A549 and H1299 after PLK1 silencing. *p < 0.05, **p < 0.01, ***p < 0.001.

To clarify the specific role of PLK1 in the development of LA, we performed further experimental validation. We detected PLK1 expression by RT-qPCR in normal lung epithelial cells Base-2b, and lung adenocarcinoma cell lines A549 and NCI-H1299, and showed that PLK1 was greatly more highly expressed in lung adenocarcinoma cells than in normal cells (*p* < 0.001) (Fig. 11A). Subsequently, We examined changes in absorbance at different time points following silencing and overexpression of the three groups of cells and then assessed cell survival using the CCK-8 kit. The results showed that the absorbance of A549 and H1299 cell lines in the overexpression group was significantly increased (*p* < 0.001) at 24, 48 and 72 H compared with the cells in the normal group (Figs. 11B and 11C), suggesting that tumor cells proliferated significantly after overexpression of PLK1. Meanwhile, the absorbance of LA cell lines in the silenced group was significantly decreased (*p* < 0.001) at 24, 48, and 72 H compared with the normal group (Figs. 11D and 11E), indicating that the proliferation ability of A549 and H1299 cell lines was significantly reduced after silencing PLK1 gene. Comparing the silencing and overexpression groups, the cells in the overexpression group showed differences but the differences were relatively insignificant, so we subsequently selected the silencing group for subsequent experiments. To verify the silencing efficiency, we performed protein blotting analysis and demonstrated that PLK1 silencing resulted in significantly lower expression compared to the normal group (*p* < 0.05) (Fig. 11F). Next, we used Annexin V method for flow cytometry to detect the apoptosis rate of A549 and H1299 after silencing PLK1, and each group was repeated three times. The results showed with that after silencing, the apoptosis rate of A549 and H1299 increased significantly compared with the normal cell group, where the HA549 cell line showed a significant increase in apoptosis after SiRNA-1 and SiRNA-2 transfection compared with the previous one (*p* < 0.05), while the H1299 cell line only showed a significant difference after SiRNA-2 transfection compared with the previous one (*p* < 0.05) (Figs. 11G–11I). Due to the higher silencing efficiency of SiRNA-2, we subsequently selected SiRNA-2 for the experiment. Thereafter, we performed protein blotting to analyze the expression of RIPK3, RIPK1, and MLKL, proteins related to necrotic apoptotic pathway, and demonstrated that the expression of RIPK3, RIPK1, and MLKL were significantly higher (*p* < 0.05) after PLK1 silencing compared to the previous one (Figs. 11J–11L).

**Figure 12:**
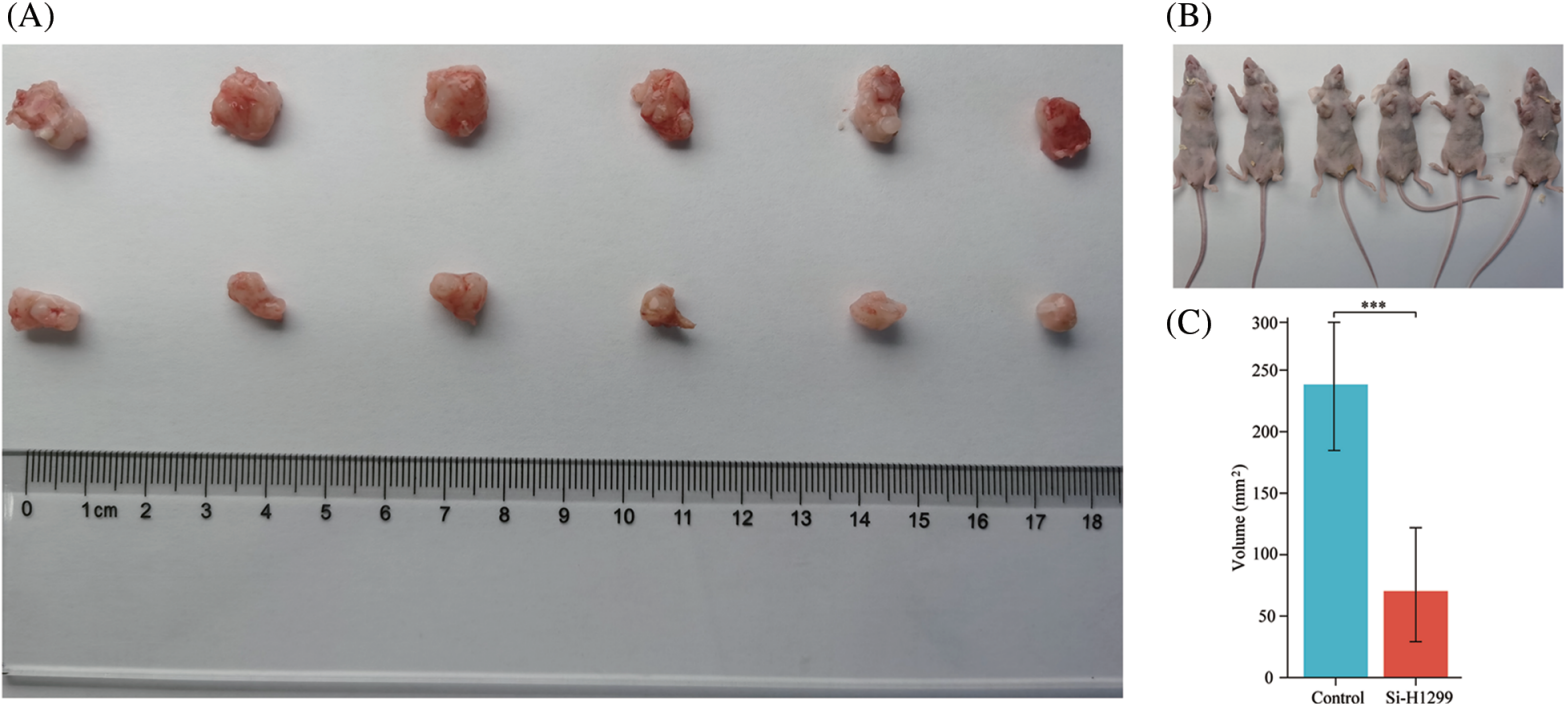
Nude mice transplantable tumor. (A, B) A549 and si-H1299 transplanted tumor size; (C) variance analysis revealed that the control group exhibited significantly larger tumor volumes compared to the experimental group (*p* < 0.001). ***p < 0.001.

To test our hypothesis, nude mice were injected subcutaneously with knockdown of PLK1 in H1299 cells and normal H1299 cells. When compared to the transplanted tumor volume found in control group, the transplanted tumor volume found in Si-H1299 group (71.50 ± 52.08 mm^3^) was significantly lower (232.33 ± 67.25 mm^3^) (*p* < 0.001) (Figs. 12A–12C), which proved that PLK1 could promote the proliferation of LA, which was also confirmed by our study.

## Discussion

PLK1 has emerged as a promising therapeutic target for lung adenocarcinoma due to its role in tumor growth, progression, and treatment resistance [29]. Preclinical studies using PLK1 inhibitors have demonstrated their efficacy in inhibiting tumor growth, inducing apoptosis, and sensitizing lung adenocarcinoma cells to conventional treatments. Furthermore, PLK1 inhibition has been shown to modulate immune responses by regulating immune checkpoint molecules such as PD-L1, suggesting its potential in enhancing the efficacy of immune checkpoint blockade therapy [30]. Targeting PLK1 holds promise for overcoming treatment resistance and improving outcomes for patients with lung adenocarcinoma. However, Several PLK1 inhibitors targeting breast and colorectal cancers have entered clinical trials recently but have demonstrated weak antitumor capacity against solid tumors [31], and few studies have focused on the therapeutic role of PLK1 in LA. Therefore, further research is needed to explore the molecular mechanisms underlying the anti-tumor effects of targeting PLK1.

To further understand the functional significance of PLK1, we conducted GO/KEGG functional enrichment analysis and identified several pathways involved in mitotic cycles, serine hydrolase pathway, neuroactive ligand-receptor interaction, and drug metabolism-cytochrome P450 pathway. The serine hydrolase pathway is a pivotal regulator of immune response, inflammation, as well as neovascularization, which have been implicated in tumor immune activity. The neuroactive ligand-receptor interaction pathway enrichment suggests a potential role of PLK1 in neurotransmission and intercellular communication. Additionally, the drug metabolism-cytochrome P450 pathway enrichment highlights the possibility of PLK1 as a drug target. Furthermore, our GSEA analysis showed that PLK1 is involved in regulating the TP53 transcription factor. Previous studies have demonstrated that PLK1 promotes tumor growth and alters the immune microenvironment by inhibiting TP53 expression and promoting apoptosis [32,33]. Therefore, PLK1 is a valuable potential therapeutic target. Currently, PLK1 inhibitors mainly target mitosis and are predominantly used for breast and colon cancers, with no clinical benefits reported yet [34]. Given the unclear mechanism of action of PLK1 in necroptosis, exploring the role of PLK1 in LA is highly valuable and provides a direction for developing new drugs.

Additionally, we found that hub genes identified through PPI analysis were predominantly members of the GAGE gene family, which primarily functions in anti-apoptosis and tumor metastasis, indicating a potential antagonistic effect of PLK1 in necroptosis. Subsequent correlation analysis demonstrated that PLK1 had negative correlation with the upstream necroptotic protein, RIPK3, validating our hypothesis. Previous studies have shown [35,36] that many cancer cells inhibit necroptosis through epigenetic silencing of RIPK3. In contrast, Park et al. [37] found that RIPK3 expression was significantly and positively correlated with tumor-infiltrating immune cell populations in various tumor types, thereby activating anti-tumor immune responses.

Therefore, considering our bioinformatic findings, we propose that PLK1 affects the level of immune infiltration in LA through necroptosis, ultimately affecting tumor proliferation. Interestingly, our study uncovers a potential interplay between PLK1, necroptosis, and the immune microenvironment. Necroptosis, known to be associated with the release of damage-associated molecular patterns (DAMPs), has been implicated in stimulating immune responses and promoting inflammation. Consequently, by modulating necroptosis [38]. PLK1 may exert an influence on the immune microenvironment and impact anti-tumor immune responses. Therefore, further investigations are warranted to comprehensively elucidate the intricate relationship between PLK1 and necroptosis, particularly within the context of cancer. Unraveling the mechanisms by which PLK1 regulates necroptosis and its implications for tumor development, treatment response, and the immune microenvironment holds promise in paving the way for novel therapeutic strategies targeting PLK1 in cancer.

Moreover, our study demonstrated a positive correlation between high expression of PLK1 and Th2 cells as well as Treg cells, while a negative correlation was observed with CD8+ T cells and B cells. Th2 cells have been reported to promote tumor progression by releasing inflammatory factors such as IL-4/IL-13, and by promoting M2 macrophage polarization and activation of myeloid-derived suppressor cells (MDSCs) [39]. Additionally, both Th2 and Treg cells have been implicated in creating an immunosuppressive microenvironment that facilitates tumor progression [40]. Furthermore, the regulation of the necroptosis pathway, which is closely associated with the inflammatory environment induced by IL-4/IL-13 [41], may be involved in PLK1-mediated modulation of the immune microenvironment in lung adenocarcinoma (LA) patients. This observation suggests that PLK1 may play a role in shaping the immune microenvironment through the regulation of Th2 and Treg cell-mediated responses, as well as through the modulation of necroptosis pathway. Further investigations are warranted to elucidate the precise mechanisms underlying PLK1’s involvement in these processes and its potential as a therapeutic target for LA by modulating the immune microenvironment. Additionally, multivariable Cox regression analysis demonstrated that PLK1 is an independent prognostic factor in lung adenocarcinoma (LA). Furthermore, the nomogram analysis also indicated that PLK1 has a favorable prognostic effect. These findings provide novel insights for the clinical assessment of patient prognosis and underscore the potential research value of PLK1.

In the experimental validation, we acquired that PLK1 promotes LA cell proliferation. Silencing PLK1 resulted in a significant increase in LA cell apoptosis, particularly in the ratio of late apoptosis to necrosis, indicating a potential inhibitory effect of PLK1 on immune infiltration through inhibiting necroptosis. WB assays further supported this speculation, as the expression of necroptotic RIPK1/RIPK3-MLKL pathway proteins was higher after PLK1 silencing than before. Additionally, the role of PLK1 in LA cell proliferation was confirmed through nude mouse transplantation tumor experiments, where tumor growth was significantly slower after PLK1 silencing than before. These findings suggest that PLK1 might regulate the tumor immune microenvironment and accelerate tumor growth by inhibiting necroptosis.

In summary, we found that PLK1 is one independent prognostic factor in LA and is closely associated with unfavorable prognostic factors in LA patients. And PLK1 may have a role in promoting tumor proliferation by reducing the level of LA immune infiltration through inhibition of necroptosis.

## Supplementary Materials

Supplementary Figure 1 Clinical correlation between PLK1 and LALA. A–C. TNM stage; D. Pathologic stage; E. Residual tumor; F. Smoker. *p < 0.05, **p < 0.01, ***p < 0.001.

Supplementary Figure 2 Correlation analysis. A. Correlation between PLK1 and immune checkpoints. B. Correlation between PLK1 and RIPK3. *p < 0.05, **p < 0.01, ***p < 0.001.

Supplementary Table 1 Baseline information table

Supplementary Table 2 Abs(log2FoldChange) top 500 differential genes

Supplementary Table 3 GO enrichment analysis

Supplementary Table 4 KEGG enrichment analysis

Supplementary Table 5 Univariate Cox regression analysis

Supplementary Table 6 Multivariate Cox regression analysis

Supplementary Table 7 Correlation of infiltrating immune

## Data Availability

The data generated for this study are available from the authors on request.

## Supplementary Materials

The supplementary material is available online at https://doi.org/10.32604/or.2023.030933.
