# Supplementary figures and images for: Polo-like kinase 1 suppresses lung adenocarcinoma immunity through necroptosis
Source: Oncol Res. 2023 Sep 15;31(6):937–53. doi: 10.32604/or.2023.030933 (PMC10513947; doi:10.32604/or.2023.030933)

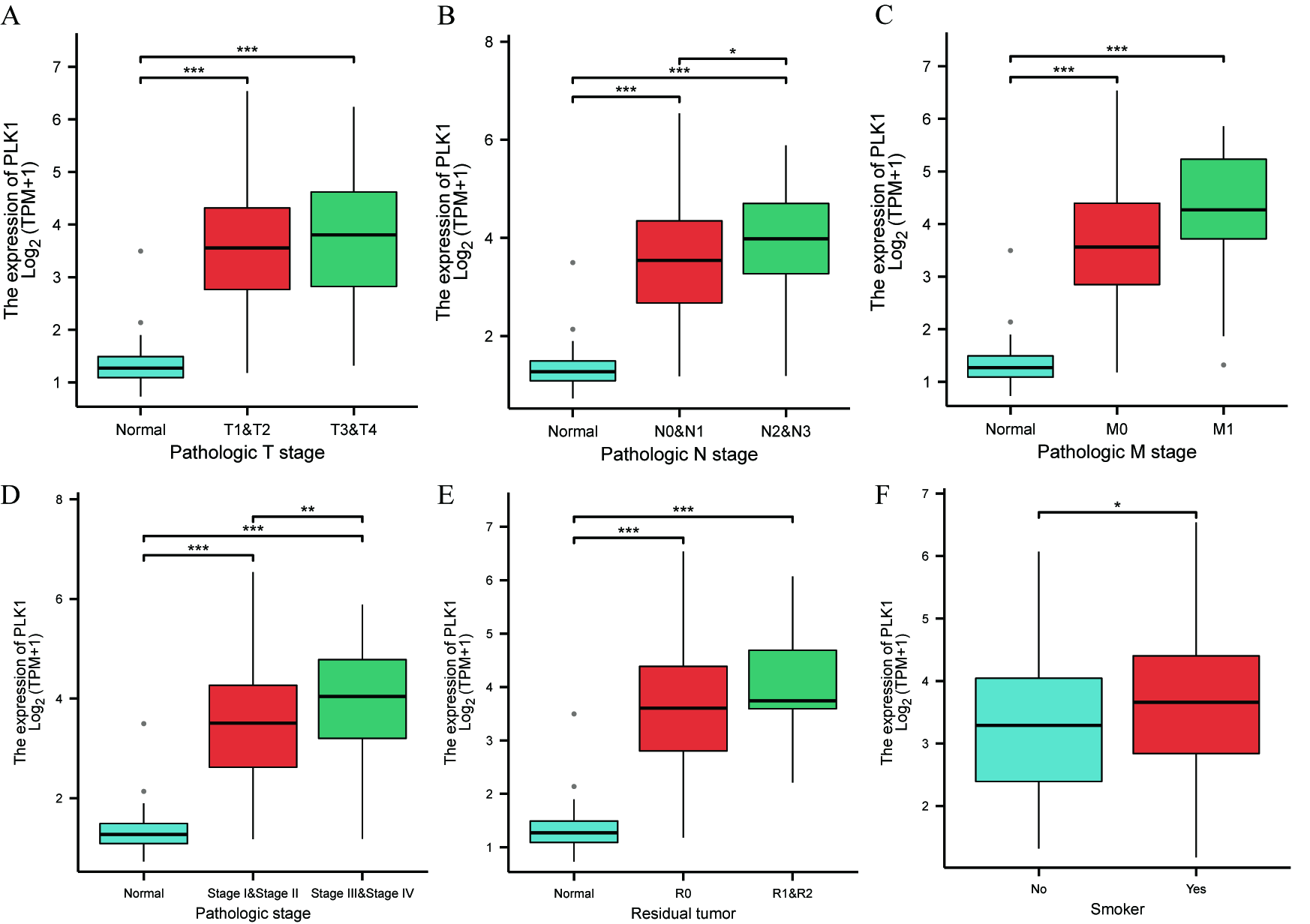

Supplement: Supplementary Figure 1 [file OncolRes-31-30933-s001.tif]

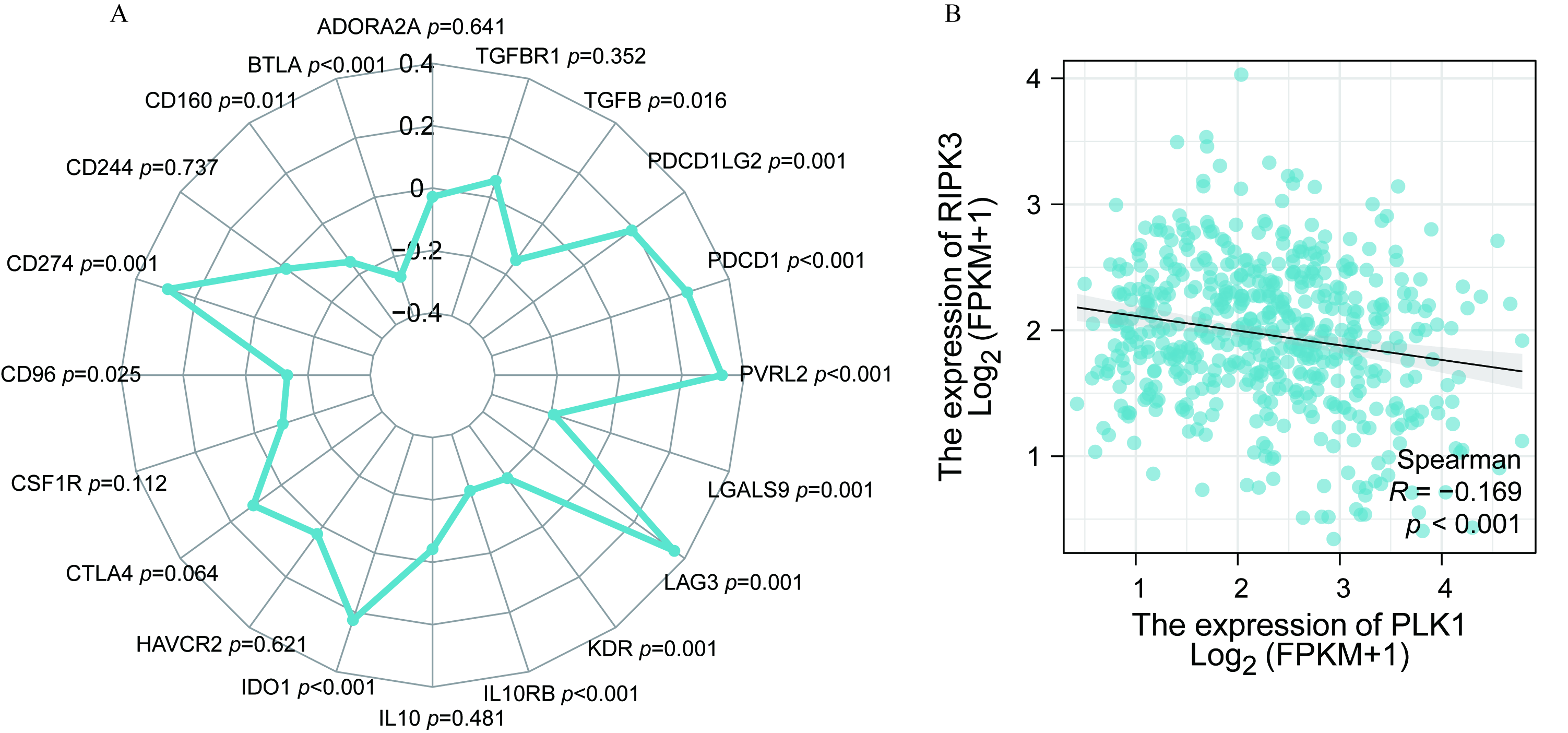

Supplement: Supplementary Figure 2 [file OncolRes-31-30933-s002.tif]
